# Lower adherence to a prudent dietary pattern is associated with earlier age at menarche in adolescents from the Growth and Obesity Chilean Cohort Study

**DOI:** 10.3389/fpubh.2022.995593

**Published:** 2023-01-30

**Authors:** Angela Martínez-Arroyo, Erika Cantor, Regina Mara Fisberg, Camila Corvalán

**Affiliations:** ^1^Food Behavior Research Center (CEIC), Faculty of Pharmacy, School of Nutrition and Dietetics, University of Valparaíso, Valparaíso, Chile; ^2^Institute of Statistics, University of Valparaiso, Valparaíso, Chile; ^3^Department of Nutrition, School of Public Health, University of São Paulo, São Paulo, Brazil; ^4^Institute of Nutrition and Food Technology (INTA), University of Chile, Santiago, Chile

**Keywords:** dietary patterns, age at menarche, puberty, adolescents, nutrition, exploratory factor analysis

## Abstract

**Introduction:**

Early menarche is associated with obesity, and metabolic and mental health risks, among other diseases. Thus, it is relevant to identify modifiable risk factors of early menarche. Some nutrients and foods have been linked to pubertal timing, but how menarche relates to overall dietary patterns is unclear.

**Methods:**

The aim of this study was to analyze the association between dietary patterns and age at menarche in a prospective cohort of Chilean girls from low and middle-income families. We conducted a survival analysis of 215 girls (median = 12.7 years, IQR = 12.2–13.2) from the Growth and Obesity Cohort Study (GOCS) who had been followed prospectively since 4 years of age (2006). Age at menarche and anthropometric measurements were recorded every 6 months since 7 years of age while diet (24-hour dietary recall) was collected for 11 years. Dietary patterns were obtained from exploratory factor analysis. Accelerated Failure Time models adjusted for potential confounding variables were used to study the association between dietary patterns and age at menarche.

**Results:**

Girls' median age at menarche was 12.7 years. Three dietary patterns were identified: “Breakfast/Light Dinner,” “Prudent” and “Snacking” which explained 19.5% of the diet variation. Girls in the lowest tertile of the “Prudent” pattern had menarche 3 months earlier than girls in the highest tertile (β: 0.022; 95% CI: 0.003; 0.041). “Breakfast/Light Dinner” and “Snacking” patterns were not associated with age at menarche.

**Conclusion:**

Our results suggest that healthier dietary patterns during puberty might be associated with menarche timing. Nevertheless, further studies are required to confirm this result and to clarify the association between diet and puberty.

## 1. Introduction

Puberty is a critical period for growth and development. Age at menarche, a later marker of puberty, is one important reproductive event related to women's health in later stages (1). Early menarche has been associated with a higher risk of obesity (2, 3), metabolic diseases (4), some forms of cancers (5), and cardiovascular diseases (6), among other health conditions (1). Although menarche has a strong genetic component, recent trends of acceleration of age at menarche show that pubertal markers are also dependable on environmental factors (i.e., education, occupation, income) (7, 8).

Some nutritional factors have been linked with the timing of puberty (7, 9). Rapid weight gain during childhood as well as obesity has been related to early age at menarche (2, 3). Additionally, consuming animal and vegetable protein during childhood might be differentially related to pubertal timing (10–12). However, the role of the food sources of animal proteins (i.e., dairy products versus red meat consumption) needs to be clarified (13–15). Recently, the consumption of non-core food such as sugar-sweetened beverages (SSBs) has also been associated with earlier age at menarche in US girls (16). Nevertheless, another study observed this association only with caffeinated and artificially sweetened beverages (17).

Dietary pattern analysis, rather than individual nutrients or foods intake, allow capturing food interactions under real-life conditions, to increase understanding on the effects of diet in health (18, 19). Nonetheless, evidence of dietary patterns and age at menarche is still scarce. A cross-sectional study conducted on Korean girls found that a “Shellfish and Processed meat” pattern was positively associated with sexual maturation (20). Another population-based study in Shanghai girls found that “Unhealthy” dietary patterns were associated with precocious puberty (21). Lastly, a prospective study on 263 Mexican girls showed that a “Vegetables and Lean proteins” pattern was associated with delayed breast development. Still, researchers found no association with age at menarche (22).

In Latin American countries, rapid changes in socioeconomic conditions, urbanization, retail foods, and public transportation have contributed to the emergence of an epidemic of childhood obesity. The nutrition transition has caused a dietary change reflecting increased consumption of animal products, ultra-processed foods, and SSBs. In contrast, the intake of vegetables, fruit, and legumes has decreased in the past decades in several countries in the region (23). The role that these dietary changes play in growth and development is still to be explored.

In Chile, the first study on menarche's age was published in 1886 and reported a mean age of 16 (24). In 2004, another cross-sectional study that included 758 girls aged 5.8–16.1 years indicated that the mean age at menarche was 12.7 years (25). Since 2006 we have been following a cohort of low-middle-income girls in the Growth and Obesity Chilean Cohort Study (GOCS) to better understand growth and development (26–30). In a previous study, we have explored the relationship between dairy consumption in childhood and pubertal development (mammary gland and age at menarche), because milk and dairy products have been related to higher growth-hormone concentrations that may affect puberty (31). However, we have not explored how the complexity of an overall diet (that considers the correlation between nutrients, non-nutrients, and foods) can influence age at menarche. Therefore, this study aimed to assess the association between dietary patterns and age at menarche in a prospective cohort of Chilean girls.

## 2. Methods

### 2.1. Study populations

Growth and Obesity Chilean Cohort Study is a population-based ambispective cohort of 1,195 children of middle-low-income families from public nursery schools, who were born in 2002–2003 in six counties from the Southeast area of Santiago, Chile. Participants were singletons who were born at term, had a birth weight ≥2,500 and < 4,500 g, and were free from conditions that could affect growth. The details of the study design, protocol, and baseline were described elsewhere (30). From the total of 1,195 children, 602 girls provided follow-up information during childhood and puberty and were considered for the current study. Repeated anthropometric and sexual maturation measurements were collected in the GOCS participants every 6 months from 7 years of age (2009). The dietary intake of participants was collected on a 6-month basis in clinic visits with a 24-h dietary recall (24HR) since 11 years of age (2013). Sociodemographic and maternal characteristics were collected by trained research assistants through a structured questionnaire.

For the present study, the identification of dietary patterns was performed using data from 458 girls who had at least one 24HR (60% had also a second 24HR) and anthropometric measurements. However, for the primary purpose of this prospective analysis, girls who completed 24HR after their first menses (*n* = 238), and girls who had not experienced menarche at the time of the present analysis (*n* = 5) were excluded. Therefore, a final sample size of 215 girls was considered for the study (Supplementary Figure S1).

### 2.2. Exposure: Dietary intake

Dietary intake data were collected on non-consecutive days by trained dietitians, face-to-face with adolescents, and the parent/caregiver responsible for preparing the meals. Multiple Pass Method (32), and a photographic atlas from the National Survey of Food Consumption of Chile were used to enable complete 24HR (33). Household measures (i.e., cups, glass, spoons) reported in each 24HR were converted into units of weight or volume, as per the standardization and quantification of the foods.

The Nutrition Data System for Research (NDSR) software (version of 2014, NCC, University of Minnesota, Minneapolis) was used to type each food and beverage of the 24HR. This program uses the United States Department of Agriculture (USDA) database as the main food composition table. For this reason, all foods and beverages reported on the 24HR were matched in the NDS-R database through the general description (i.e., name, type, and mode of preparation). Energy and macronutrient values available in Chilean food composition tables (34), local food industry composition tables, and/or nutrition labels were required (80 and 120% for each parameter) to accept a food selection. Regional foods and traditional dishes commonly consumed (i.e., *porotos con riendas, pastel de choclo*) were also inserted into the program.

A total of 771 different food items were reported in each 24HR and 732 were consumed by at least 5% of the sample. These food items were classified into 30 food categories based on their nutritional value, the correlation coefficient between groups, communalities values, food preparation, and Chilean dietary behaviors. In addition, evidence of potentially relevant food groups for pubertal development (i.e., whole-milk, low-fat milk and non-fat milk, yogurt, red meat, poultry, fish, processed meat, and soft drinks) were considered for the study (Supplementary Table S1).

### 2.3. Outcome: Age at menarche

Starting at 7 years old, a single pediatric endocrinologist assessed pubertal development in girls based on the Tanner scale (35). Age at menarche was prospectively collected by self-report. Starting at breast Tanner stage 4, mothers and girls were advised to call researchers to inform menarche, and telephonic follow-ups were performed every 6 months. A questionnaire was developed to differentiate vaginal infections or other genitourinary conditions from first menses (36). Age at menarche was considered as the time in months from birth to the age of first menses, and the outcome was defined as attaining menarche.

### 2.4. Covariates

Weight and height were measured closer to the age at menarche with the use of standardized techniques by trained dietitians as described elsewhere and were evaluated in this study (37). Body mass index (BMI) was calculated using the standardized formula of weight (kg) divided by height squared (m^2^) and BMI *z*-scores were calculated based on the age of the participants according to the World Health Organization Growth Reference (38)(WHO). The girls' body weight status was classified as underweight (≤ −1 SD), normal weight (< −1 SD and < +1 SD), overweight (≥ +1 SD and < +2 SD), and obesity (≥ +2 SD).

Participants' mothers self-reported information on education level (dichotomized as ≥12 or < 12 years) and age at menarche were collected *via* a structured questionnaire. A trained dietitian also measured the mother's current height and weight to calculate BMI according to WHO cutoffs for adults (39). Time spent on TV during the days of the week and weekend (≥2 or < 2 h/day) for adolescents, hours of sleep (≥9 or < 9 h/day), and attendance on the School Feeding Program (yes or no) were provided from mothers at clinic visits using a semi-structured questionnaire.

We estimate the usual energy intake, adjusting for intra-individual variance according to the Multiple Source Method (40). In addition, we calculated misreporting of energy intake according to the equation: EI (energy intake)-EER (estimated energy requirements)/EER × 100 (41).

### 2.5. Statistical analysis

#### 2.5.1. Dietary patterns

The dietary patterns of the GOCS participants have already been reported elsewhere (42). Because, the literature suggests that there are dietary sex differences (43) since girls may have more structured feeding than boys (i.e., girls are more concerned about body image) (44), dietary patterns were re-estimated for the purpose of the current study.

Initially, statistical modeling techniques incorporated into the Multiple Source Method, an online platform, were used to estimate the usual intake of each food group in grams (40). Exploratory factor analysis (EFA) was used to identify the dietary patterns from the 30 food groups. Factors with eigenvalues ≥1.0 were retained. In the second stage, the scree plot was visually inspected, determining that three factors should be retained (45). We used Kaiser–Meyer–Olkin (KMO) test and Bartlett's sphericity test to evaluate sample adequacy (46). Varimax orthogonal rotation was applied to improve the interpretability of the factor loading matrix and to ensure the independence of the factors derived. Factor loadings ≥|0.30| were considered to contribute to each pattern. The dietary patterns were named according to their interpretability, the characteristics of the food in each dietary pattern, and the communalities observed, which reflect the level of linkage between the variable (food group) and the extracted factor. Other food grouping systems were tried but they did not allow us to create dietary patterns, due to low or across factor loading and their interpretability (45). For each participant, a factor score of each dietary pattern was calculated using the regression scoring method (47). The dietary pattern scores were categorized into tertiles and used as exposure variables.

#### 2.5.2. Diet and age at menarche models

Cox regression models were used to evaluate the relationship between the exposure variables and the age at menarche. However, these models violated the proportional hazards assumption, which was tested graphically using a plot of the log cumulative hazard using ln{–ln[S(t)]}. Consequently, Accelerated Failure Time models (AFT) were used to evaluate the relationship between dietary patterns and age at menarche. The exponential AFT model, the Weibull AFT model, the log-logistic AFT model, and the log-normal AFT model were fitted to the data set. The Bayesian Information Criterion, Akaike Information Criterion, and Cox-Snell residuals were used to evaluate the performance of each model. Finally, the log-logistic AFT model was chosen as the best fitted model. The Multivariate AFT model was estimated using the backward stepwise procedure including those variables with *p*-value < 0.10 in the univariate analysis.

For sensitivity analysis, the multivariate model was adjusted by usual energy intake [two 24HR at least 60% sample, through Multiple Source Method (40)] and misreporting energy intake which could be considered as confounders of the associations with dietary patterns. A *p*-value < 0.05 was considered statistically significant. All the analyses were made using Stata 16^®^ (StataCorp., College Station, Texas, USA).

## 3. Results

The estimated median age at menarche was 12.7 years old (IQR = 12.2–13.2). Almost half of the participants (43.2%) presented excess weight (overweight and obesity). One out of three mothers reported < 12 years of education, about one-third had obesity and at least one out of three self-reported age at menarche < 12 years old (Table 1).

**Table 1:** Anthropometric, sociodemographic, and maternal characteristics of 215 girls of the Growth and Obesity Chilean Cohort Study 2013–2017.

	***n***	
**Total girls**	215	
Age at first 24HR visit, y^a^	215	11.6 (11.3–11.9)
Age at tanner stage 4 visit, y^a^	215	12.3 (11.8–12.9)
Weight before menarche, kg^a^	215	46.8 (45.3–57.9)
Height before menarche, cm^b^	215	152.6 (± 6.1)
BMI-for-age *z* score before menarche^b^, ^c^	215	0.68 (± 1.2)
**Weight status (%)** ^e^		
Under weight	17	7.9
Normal	105	48.8
Overweight	62	28.8
Obesity	31	14.4
**Sleep (hours)**^d^ **(%)**		
< 9 h	129	67.9
≥9 h	61	32.1
**Television watching (hours/day)**^d^ **(%)**		
< 2 h	120	69.4
≥2 h	53	30.6
**School feeding program (%)**		
Yes	88	40.9
No	127	59.1
**Maternal variables**		
**Age at menarche (years)**^a^, ^d^	187	13.0 (12.0–14.0)
**Weight status (BMI) (%)**^d^, ^f^		
Normal	41	19.5
Overweight	92	43.8
Obesity	77	36.7
**Schooling (years)**^d^ **(%)**		
< 12 years	74	34.7
≥12 years	139	65.3

^a^Values are medians and IQR in parentheses.

^b^Values are mean, SD in parentheses.

^c^BMI, Body mass index.

^d^Number missing data: sleep, 25; Television watching, 42; maternal age at menarche, 28; maternal weight status, 5; maternal schooling, 2.

^e^Weight status, BMI-for-age z score: underweight ≤ −1 SD, normal >−1 SD and < 1 SD, overweight ≥+1 SD and < +2 SD, obesity ≥+2 SD.

^f^Maternal weight status: normal < 25 kg/m^2^, overweight ≥25 to < 30, obesity ≥30 kg/m^2^.

Exploratory factor analysis allowed the identification of three dietary patterns: (i) “Breakfast/Light Dinner” consisting of tea, sugar, bread, margarine/butter, and cold cuts (positive loadings) and flavored milk (negative loading); (ii) “Prudent” consisting of white meats (poultry and fish), fruits, vegetables, homemade salad dressing (i.e., oil, lemon, salt, and vinegar) (positive loadings) and processed meats (negative loadings); (iii) “Snacking” consisting of soft drinks, junk food, cookies and cakes, and soups (positive loadings) and yogurt, and ready-to-eat cereal (negative loadings). These patterns explained variance in total intake of 7%, 6.4%, and 6% (Table 2).

**Table 2:** Factor loadings^a^, explained variance and eigenvalues of the three major dietary patterns practiced by girls from the Growth and Obesity Chilean Cohort Study^b^.

**Food groups**	**Breakfast/** **light dinner**	**Prudent**	**Snacking**
Low fat milk	−0.14	0.07	0.09
Whole fat milk	−0.04	0.12	0.07
Yogurt	−0.10	−0.03	**−0.64**
Cheeses	0.26	−0.19	−0.14
Flavored milk	**−0.31**	−0.22	−0.14
Red meat	0.07	0.18	−0.08
Lean meats	−0.09	**0.34**	0.13
Processed meats	0.08	**−0.33**	0.06
Cold cuts	**0.43**	−0.14	−0.21
Junk food	−0.23	−0.13	**0.34**
Flavored juices	−0.21	0.09	0.04
Soft drink	−0.01	−0.16	**0.37**
Tea and coffee	**0.64**	0.07	0.26
Bread	**0.72**	−0.04	−0.14
Ready to eat cereal	−0.10	−0.08	**−0.61**
Rice, pastas, potatoes	0.16	−0.05	−0.10
Vegetables	0.06	**0.73**	0.04
Fresh fruit	0.02	**0.41**	−0.17
Eggs	−0.02	0.18	−0.16
Homemade dishes	0.00	0.20	−0.09
Cracker and salt snack	−0.12	−0.20	0.18
Confectionery and chocolate	−0.04	−0.06	0.16
Cookies and cake	−0.21	−0.07	**0.40**
Desserts and ice cream	0.11	0.18	0.02
Sugar	**0.56**	0.20	**0.34**
Chocolate powder	−0.26	0.15	0.03
Margarine and butter	**0.52**	−0.10	0.00
Homemade salad dressing	−0.03	**0.65**	0.06
Mayonnaise and ketchup	0.10	0.07	0.04
Soups	−0.05	0.16	**0.33**
% of explained variance	0.07	0.06	0.06
% of accumulated explained variance	0.07	0.14	0.19
Eigenvalues	2.22	1.89	1.70

^a^Factor loadings > |0.30| are shown in bold for readability purposes. Bartlet sphericity test (BSS) = p < 0.0001. Kaiser–Meyer–Olkin = 0.54.

^b^Exploratory Factor Analysis in the total sample girls GOCS of n = 458 girls.

In the univariate analyses, the “Prudent” pattern was significantly associated with the age at menarche (*p* < 0.04). In addition, girls grouped in the second and third tertile of the prudent pattern had a later age of menarche compared to those classified in the first tertile (Table 3). Girls who were overweight or obesity status experienced menarche earlier than those who were underweight or normal weight, suggesting an inverse relationship between age at menarche and weight status (Table 3). Age at menarche did not vary significantly with respect to school feeding program, hours of sleep, hours of television, “breakfast” pattern, “snacking” pattern, and other maternal characteristics.

**Table 3:** Univariate comparison of the age at menarche according to anthropometric characteristics, dietary patterns, and maternal characteristics of 215 girls from the Growth and Obesity Chilean Cohort Study.

	***N***	**Age at menarche^a^**	***P-*values^e^**
**Weight status** ^b^			
Under weight	17	13.2 (12.6–13.6)	
Normal	105	12.7 (12.3–13.3)	0.006
Overweight	62	12.4 (12.0–12.9)	
Obesity	31	12.6 (12.1–12.9)	
**School feeding program**			
Yes	88	12.7 (12.2–13.2)	0.91
No	127	12.6 (12.2–13.2)	
**Sleep (hours)** ^c^			
< 9 h	129	12.7 (12.1–13.3)	0.63
≥9 h	61	12.7 (12.3–13.0)	
**Television watching (hours/day)** ^c^			
< 2 h	120	12.7 (12.3–13.2)	0.982
≥2 h	53	12.6 (12.2–13.4)	
**“Breakfast/Light dinner” pattern**			
T1	77	12.7 (12.1–13.2)	
T2	69	12.7 (12.2–12.9)	0.833
T3	69	12.7 (12.3–13.3)	
**“Prudent” pattern**			
T1	72	12.5 (12.1–12.9)	0.040
T2	77	12.7 (12.3–13.3)	
T3	66	12.8 (12.3–13.2)	
**“Snacking” pattern**			
T1	71	12.6 (12.1–13.2)	0.385
T2	79	12.6 (12.2–13.1)	
T3	65	12.7 (12.3–13.4)	
**Maternal age at menarche (years)** ^a, c^			
< 11 y	37	12.7 (12.1–13.1)	0.089
12–14 y	120	12.6 (12.2–12.9)	
>15 y	30	13.0 (12.2–13.4)	
**Maternal weight status (BMI)**^c^, ^d^			
Normal	41	12.8 (12.2–13.3)	
Overweight	92	12.7 (12.2–13.2)	0.277
Obesity	77	12.6 (12.1–13.0)	
**Maternal schooling (years)** ^c^			
< 12 y	74	12.7 (12.3–13.2)	0.409
≥12 y	139	12.6 (12.1–13.2)	

^a^Values are medians and IQR in parentheses.

^b^Weight status, BMI-for-age z score: underweight ≤ -1 SD, normal >-1 SD and < +1 SD, overweight ≥+1 SD and < +2 SD, obesity ≥+2 SD.

^c^Number missing data: sleep, 25; Television watching, 42; menarche mother, 28; status weight mother, 5; maternal schooling, 2.

^d^Maternal weight status: normal < 25 kg/m^2^, overweight ≥25 to < 30, obesity ≥30 kg/m^2^.

^e^Mann–Whitney or Kruskal–Wallis test.

**Figure 1:**
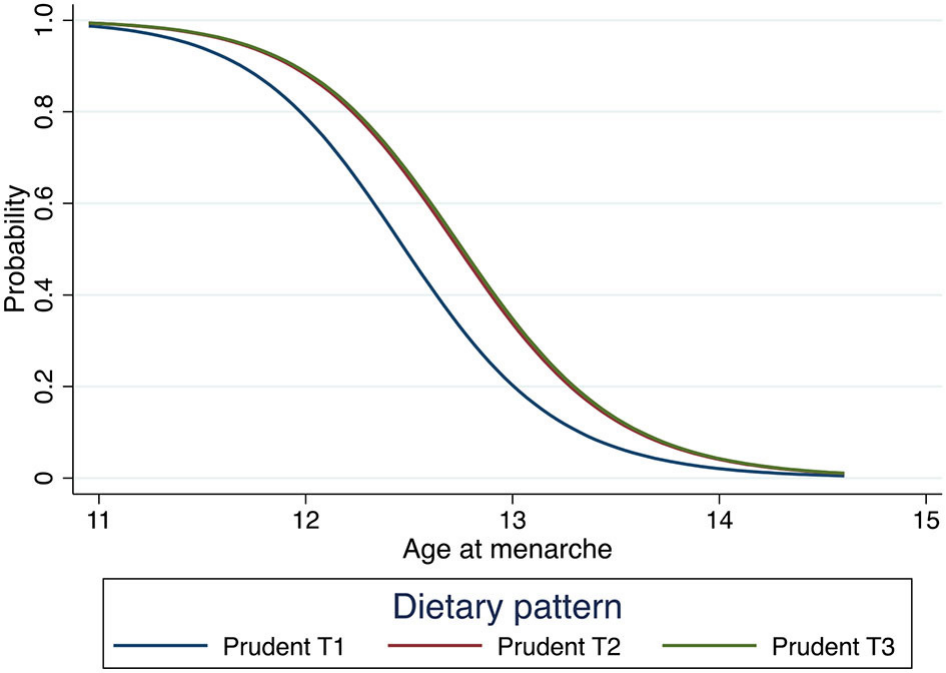
Survival curve of age at menarche of each of the tertiles of the “Prudent” dietary patterns in 215 girls from the Growth and Obesity Chilean Cohort Study. Model adjusted by *z*-score BMI maternal age at menarche and total usual energy intake.

After adjusting for BMI-for-age z scores, mothers' age at menarche, and total energy intake, we observed that girls in the lowest tertile of scores for a “Prudent” dietary pattern had on average their age at menarche 3 months earlier than those with higher adherence (ß: 0.022; 95% CI: 0.003; 0.041) after adjusting for BMI-for-age z scores and mothers' age at menarche, usual total energy intake (Table 4, Figure 1).

**Table 4:** Associations between tertiles of adherence to the “Prudent” dietary pattern and age at menarche in 215 girls from the Growth and Obesity Chilean Cohort Study.

**Prudent dietary pattern**	**Model 1^*c*^**	**Model 2^*c*^**	**Model 3^*c*^**
	**β*s*(95*%CIs*)^a^**	**β*s*(95*% CIs*)**	**β*s*(95*%CIs*)**
T1^b^	Reference	Reference	Reference
T2	0.019 (0.001; 0.037)	0.022 (0.004; 0.04)	0.021(0.002; 0.039)
T3	0.023 (0.004; 0.041)	0.021 (0.002; 0.039)	0.022 (0.003; 0.041)

^a^All values are βs (95% CIs). βs were estimated from a log-logistic AFT model.

^b^Tertile of adherence to dietary pattern.

^c^Model 1: crude, Model 2 adjusted by z-score BMI and maternal age at menarche; Model 3 was adjusted for Model 2 and usual energy intake.

## 4. Discussion

In this prospective study of Chilean adolescents, we found that girls with lower adherence to the “Prudent” dietary pattern during adolescence had slightly earlier age at menarche (three months), compared to those with higher adherence, independently of their nutritional status.

The associations described for the “Prudent” pattern are consistent with the literature that has examined individual foods and/or nutrients. Our “Prudent” dietary pattern had a higher and positive factor loading to vegetables, fruits, homemade salad dressing, and lean meats (poultry, fish) groups and negative factor loading to processed meat. Girls with lower adherence to this pattern might suggest that they have a lower intake of vegetal protein, fiber, and phytoestrogens. Although there is some consistency in studies that the intake of vegetal proteins during middle childhood has been related to later menarche, the causal mechanisms are unknown or have other components that confound your relation (7, 10, 11, 48). While a higher fiber intake is likely linked to delayed menarche onset (49) because the fiber intake might modulate circulating estrogen levels, given that it affects puberty onset mediated by the hypothalamus-pituitary–gonadal system (9). In addition, isoflavones, a phytoestrogen, which are structurally and functionally similar to endogenous estrogens, inhibiting effects on aromatase have been associated with the timing of puberty (9). Nevertheless, in previous analyses, we found a lower intake of phytoestrogens in GOCS girls, a pattern characteristic of the western diet (50). On the other hand, a higher intake of animal proteins and meats has been associated with earlier age at menarche (51) while canned tuna/ sardine intake frequency had been positively associated with age at menarche (52). Thus, we show here that it is most likely the combination of the intake of healthy foods (i.e., dietary pattern) rather than single foods intake that explains the associations previously described with age at menarche. Moreover, it is likely that associations between dietary patterns and pubertal timing reflect subjacent constructs, underlying overall diets (45).

The dietary patterns identified in girls were similar to those described for the total sample of the GOCS (42). The “Prudent” pattern was akin to the “Natural food” but with other items of healthy food, that we did not observe in the total sample GOCS, because here we tested other combinations of food groups according to factor loadings, communalities, and interpretability (42). In our cross-sectional analyses, the “Natural food” pattern was associated with overweight in the overall sample, possibly reflecting reverse causality. In this study, we adjusted for BMI-for-age *z*-score and total energy intake, and interestingly, we found that the association between the “Prudent” dietary pattern and age at menarche remained significant showing that not only the caloric content of the diet but the nature and quality of the foods are relevant at least in terms of developmental milestones. In this same cohort, we have previously described that the consumption of yogurt (>125 g/d) was associated with older age at menarche (4.6 months), however, milk or yogurt did not have positive factor loadings in the most prevalent dietary patterns that we identified, and thus, we were unable to corroborate these findings (31).

We did not find that the “Breakfast/Light Dinner” and the “Snacking” patterns which were characterized by energy-dense foods, added sugars, solid fats, and sodium, were associated with age at menarche. It has been suggested that foods with a high-glycemic index could trigger hormonal regulations and other biological mechanisms that could result in earlier age at menarche (4, 16). Moreover, tea and coffee consumption has been linked to earlier menarche (17) and both had high positive factors loading in these dietary patterns. Cross-sectional dietary patterns studies from Korea (20) and Shanghai (21) have shown a positive association between “Unhealthy” or “Shellfish and Processed meat patterns,” respectively, and puberty. However, one of the main differences with our study is that they evaluated a marker of puberty onset (development breast), highlighting those early and late markers of puberty might have different determinants. To the best of our knowledge, we only found one study that longitudinally evaluated dietary patterns and age at menarche. Duan et al. (53) found in Chinese girls, a sample with high adherence to “Modern” dietary patterns, was associated with higher odds of experiencing menarche at an earlier age. Taken together, this evidence suggests that unhealthy dietary patterns have a potential influence on pubertal timing through metabolic changes in insulin-mediated pathway mechanisms and the upregulation of hormones (21).

In the GOCS study, we have detailed information on several pubertal markers (breast bud, menarche, high peak velocity, etc.) (36). However, dietary recalls were started when many girls had already started puberty, for this reason, to maintain a prospective analysis we had to choose the age at menarche as our primary outcome. Age at menarche is one of the most used pubertal markers in epidemiological studies. Although it is easily remembered by the participants, its determination is not exempt from challenges. Girls who had their menarche before starting dietary assessments were excluded which could have potentially introduced selection bias but not affect the internal validity of the results. In our study to decrease misclassification, we collected prospective data, and we conducted a detailed questionnaire to exclude vaginal infections, vaginal wounds, or other sources of bleeding that would not correspond to the first menses. However, our study did not have information on stress levels, a well-accepted predictor of age at menarche (1).

This study also has several strengths. First, given the prospective design, we could estimate the temporality of the studied associations between peripubertal diet and age at menarche, excluding the possibility of reverse causality. Diets were recorded using a robust method of estimation that allows decreasing errors inherent to a measurement based on the respondent's memory. The “Multiple-Pass Method” was applied to certify that all foods were listed properly by the adolescents and there was no missing information. In addition, the usual intake adjusted for intra-individual variance according to the “Multiple Source Method” was estimated to increase the reliability of the results (54). Also, we did not find differences in sensitivity analysis when adjusting for misreporting of energy intake or total energy intake. Nevertheless, limitations should be noted. First, the factor exploratory analysis method has limitations due to the theoretical or optional decisions that a researcher makes which can influence the findings of the research (45). In addition, factor analysis allows the identification of dietary patterns of a particular sample, but these patterns may not necessarily represent the ideal diets since they are based on data (55). Moreover, our dietary patterns only explained about 20% of the diet variability, suggesting that there might be other patterns that could explain the remaining variability. Second, the results cannot be generalized to girls from high socio-economic levels and the sample size was based on date availability rather than power. Third, although the 24HR can provide recall bias (56), the interviews were reported together with parents/caregivers, and a complete list of foods from the School Nutrition Program was provided. Moreover, we know that diet can present measurement error derived from self-reports, however, in the present study analyses were adjusted by total usual energy intake to account for this issue (44). Finally, although a priori sample size calculation was not performed, and 215 girls could be considered a limited sample size to detect a significant association between dietary patterns and the onset of menarche, this number of girls is sufficient to estimate the model coefficients without bias and with high precision because we have ~54 events per variable (57).

In conclusion, in this prospective study of Chilean adolescents, we found that girls with lower adherence to the “Prudent” dietary pattern during adolescence had slightly earlier age at menarche (3 months), compared to those with higher adherence. Although our result of age at menarche could seem irrelevant from a clinical perspective, these changes are in the range of the results described in other population studies, both descriptive and in association with different health outcomes (49, 58–62). Based on our study results, and the existing evidence, we considered that an adequate and balanced healthy diet food based on dietary guidelines during this stage is sufficient to ensure proper growth and normal pubertal development. Further research is needed due to the lack of understanding of many mechanisms of how diet is related to puberty.

## Data availability statement

The original contributions presented in the study are included in the article/Supplementary material, further inquiries can be directed to the corresponding author.

## Ethics statement

The studies involving human participants were reviewed and approved by Ethics Committee of the Institute of Nutrition and Food Technology, University of Chile. Written informed consent to participate in this study was provided by the participants' legal guardian/next of kin.

## Author contributions

CC, AM-A, and RMF contributed to the study's conception and design. CC provided essential materials. AM-A wrote the first draft of the manuscript. EC and AM-A are involved in data analysis. AM-A, RMF, CC, and EC contributed to the interpretation of the results. All authors contributed to the manuscript writing and read and approved the final manuscript.
